# Study of the surface properties of ZnO nanocolumns used for thin-film solar cells

**DOI:** 10.3762/bjnano.8.48

**Published:** 2017-02-16

**Authors:** Neda Neykova, Jiri Stuchlik, Karel Hruska, Ales Poruba, Zdenek Remes, Ognen Pop-Georgievski

**Affiliations:** 1 Institute of Physics AS CR v.v.i., Cukrovarnicka 10, 162 53 Prague, Czech Republic; 2 Institute of Macromolecular Chemistry AS CR v.v.i., Heyrovsky sq. 2, 162 06 Prague, Czech Republic

**Keywords:** 3-dimensional solar cells, hydrothermal growth, optical spectroscopy, photothermal deflection spectroscopy, plasma treatment, X-ray photoelectron spectroscopy, ZnO nanocolumns

## Abstract

Densely packed ZnO nanocolumns (NCs), perpendicularly oriented to the fused-silica substrates were directly grown under hydrothermal conditions at 90 °C, with a growth rate of around 0.2 μm/h. The morphology of the nanostructures was visualized and analyzed by scanning electron microscopy (SEM). The surface properties of ZnO NCs and the binding state of present elements were investigated before and after different plasma treatments, typically used in plasma-enhanced CVD solar cell deposition processes, by X-ray photoelectron spectroscopy (XPS). Photothermal deflection spectroscopy (PDS) was used to investigate the optical and photoelectrical characteristics of the ZnO NCs, and the changes induced to the absorptance by the plasma treatments. A strong impact of hydrogen plasma treatment on the free-carrier and defect absorption of ZnO NCs has been directly detected in the PDS spectra. Although oxygen plasma treatment was proven to be more efficient in the surface activation of the ZnO NC, the PDS analysis showed that the plasma treatment left the optical and photoelectrical features of the ZnO NCs intact. Thus, it was proven that the selected oxygen plasma treatment can be of great benefit for the development of thin film solar cells based on ZnO NCs.

## Introduction

The widely accepted design of thin-film silicon (TF-Si) solar cells, used for mass production, is composed of a transparent conductive oxide with roughness at the nanoscale on the front (TCO), e.g., tin oxide (SnO_2_) or zinc oxide (ZnO), followed by p–i–n Si layers (amorphous and/or nanocrystalline) in the cell and a back reflector [1–2]. In such a layer arrangement, the light scattering and the consequent light trapping, caused by the interfaces with nano-scale roughness (front TCO–active layer and active layer–back reflector), increase the optical path inside in the thin silicon layer. These effects are observed in the weakly absorbing spectral region of silicon above approximately 650 nm, leading to efficiencies well above 13% at the cell level and above 12% at the module level [3–4]. However, the photo-generated current, determined by light absorption, is limited by the drift of generated electrons and holes across the absorber layer. Thus, the highest performances are expected for solar cells having a sufficiently large “optical thickness” and a sufficiently short distance between the electrodes, the “electrical thickness”. In common planar TF-Si solar cells, it is impossible to simultaneously fulfil these two conditions. Recently developed solar cells based on a three dimensional (3-D) design, in which periodically ordered zinc oxide nanocolumns (ZnO NCs) are used as a front electrode, have been of great interest, because they would exceed in the ultimate light trapping and provide excellent charge separation [5–7]. Due to the vertical geometry of these textures, the optical thickness is dictated by the height of the NCs, such that most of the light traversing the cell sees an absorber-layer thickness approximately equal to the NC height. In contrast, as the front and back TCO contacts are interpenetrating, the inter-electrode distance, given by the thickness of the Si layers on the walls of the NCs, is generally substantially thinner than that applied for state-of-the-art a-Si:H solar cells; the lateral carrier transport provided by this type of texture should thus ensure an optimal current collection. Therefore, it is envisaged that in comparison to thin-film planar cells with nano-scale roughness, the 3-D solar cells might lead to higher efficiency providing important assets such as minimal material consumption [8–10]. The proposed 3-D concept is not limited to thin-film silicon solar cells, but could be advantageously used for all other thin-film solar cells.

So far, a wide diversity of methods have been used for the preparation of ZnO nanocolumns such as metal organic chemical vapor deposition (MOCVD) [11], electrochemical deposition [12], sputtering [13], reactive ion etching [5] and the hydrothermal method [6,14–15]. The last mentioned is an attractive and preferable method for growing one-dimensional structures of ZnO, as it is simple, does not require expensive equipment, is safe and environmentally friendly since water is used as a solvent, and it is easy to scale-up for further mass production.

Solar cell deposition is a multistep process during which different plasma processes are being used. Oxygen plasma is applied for both, activation of the surface and stripping of the polymer mask used for fabrication of periodically ordered ZnO NCs, while hydrogen plasma is usually used immediately before the deposition of the active solar-cell layer for directly increasing the electrical conductivity. The employed plasma treatments could significantly influence the concentration of defects and free carriers, reflected in the defect and free-carrier adsorption, and consequently impact the efficiency of the solar cell. Therefore, the investigation of the effects of the different plasma treatments on the ZnO nanocolumns is of crucial importance.

Herein, a low-temperature hydrothermal method is used to synthesize densely packed NCs on fused silica substrates covered with a ZnO seed layer, which were prepared before by DC reactive magnetron sputtering. The optical absorption of the pristine ZnO layers as well as that of the substrates bearing the dense ZnO NCs was investigated by photothermal deflection spectroscopy (PDS) [16–17]. Furthermore, we investigated the changes in the PDS spectrum of the dense NCs induced by hydrogen and oxygen plasma treatment under conditions typical for plasma processing of thin-film silicon solar cells. XPS was used to determine the changes in surface composition as a result of the different plasma treatments.

## Results and Discussion

**Figure 1:**
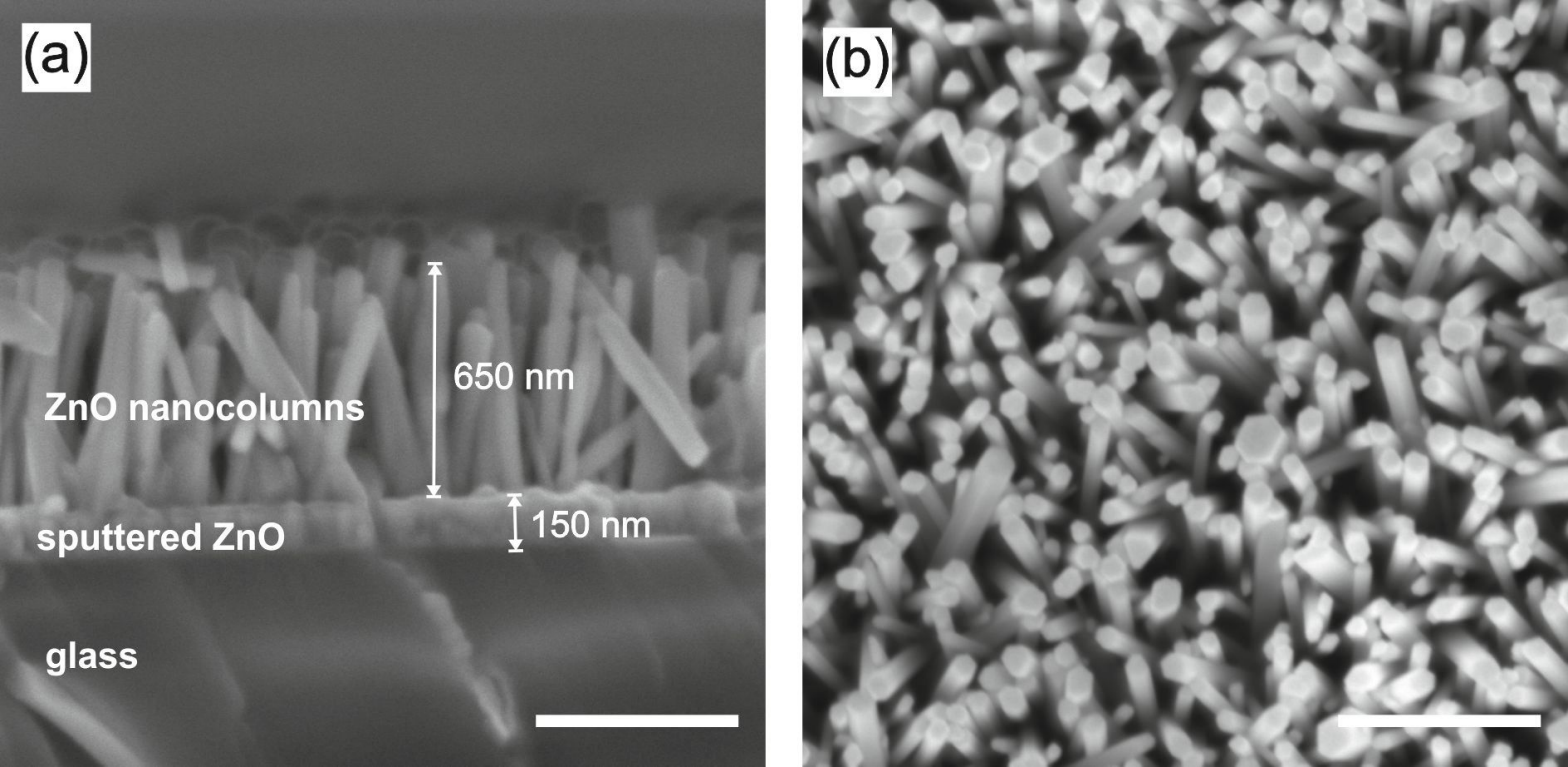
Cross-sectional (a) and top (b) view of densely packed ZnO NCs grown at 90 °C for 180 min (scale bar: 500 nm).

Figure 1 reports SEM images of densely packed ZnO nanocolumns grown at 90 °C for 180 min. As it can be seen from the SEM cross-sectional view, the ZnO nanocolumns are not interconnected and are well spaced with gaps of several nanometers. Notably, it is evident that each column has well defined boundaries (Figure 1a). The position of the individual nanocolumns is random with diameters varying from around 30 to 180 nm, as revealed by the top-view SEM image (Figure 1b).

An average nanocolumn length of 650 nm was measured from the cross section, as shown in the SEM image of Figure 1a. The thickness of the seed layer is about 150 nm. It should be noted, that after the hydrogen or oxygen surface plasma treatments the morphology of the nanocolumns does not change (see Figure S1 and Figure S2, Supporting Information File 1 for details).

**Figure 2:**
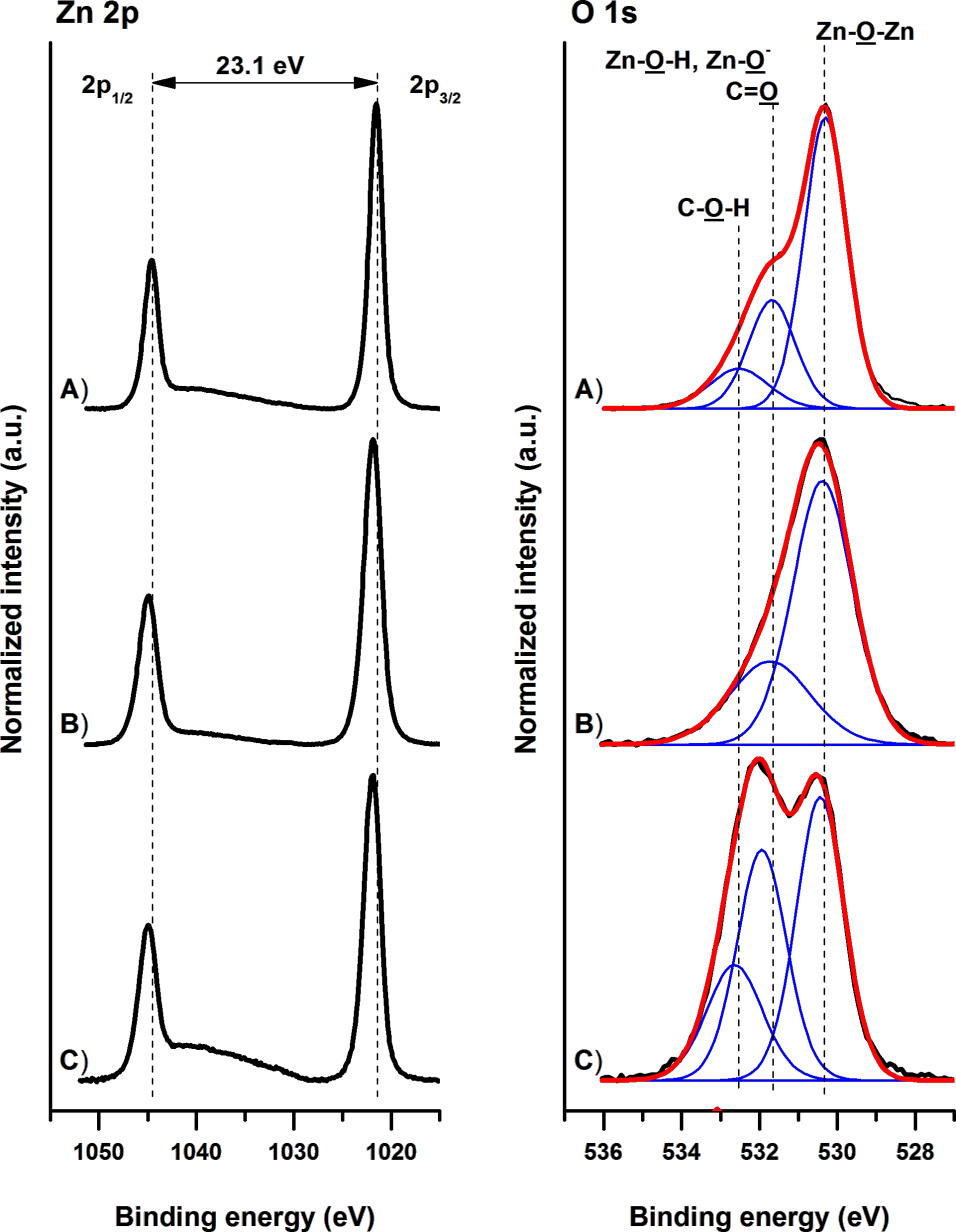
High-resolution core-level Zn 2p and O 1s XPS spectra of pristine (A) ZnO NCs and ZnO NCs after 25 min treatment in (B) H- and (C) O-plasmas. Measured spectra are presented with black lines, while their corresponding fitted envelopes are presented in red. The individual contributions of different functional groups present in the ZnO nanocolumns are represented with blue lines.

The chemical bonding structure of the ZnO films prepared by hydrothermal growth from a seed layer on fused silica carriers was examined by XPS. Figure 2 reports typical XPS high-resolution Zn 2p and O 1s spectra of the ZnO nanocolumns before and after 25 min of treatment in H- and O-plasma. The pristine ZnO NCs are characterized by a characteristic spin-split doublet with a Zn 2p_3/2_ peak centered at 1021.6 eV and a Zn 2p_1/2_ peak (at 1044.7 eV) showing 23.1 eV separation from the main contribution. The O 1s spectrum of the as-prepared nanocolumns could be deconvoluted with three contributions arising from lattice oxygen (Zn–O–Zn), non-lattice oxygen (Zn–O–H, Zn–O^−^) and O=C moieties, and C–O–H contributions centered at 530.4, 531.7 and 532.5 eV, respectively. The O=C and C–O–H contributions originate from organic contaminants adsorbed on the ZnO NCs surface during the transfer of the substrates to the XPS chamber.

The plasma treatments that are typically used in the solar cell fabrication processes, shift the position of the Zn 2p peaks by 0.3–0.4 eV toward higher binding energies and significantly broaden their width, irrespectively of the exposure time. While the full width at half maximum (FWHM) of the pristine nanocolumns is about 1.8 eV, the FWHM of H- and O-plasma treated ZnO structures increased to 2.3 and 2.0 eV, respectively. The observed changes in the high-resolution Zn 2p spectra indicate the enhanced presence of surface defects, i.e., the existence of Zn atoms in different chemical surroundings. Concomitantly, the plasma treatment introduces changes in the high-resolution O 1s spectra and a strong increase of the contributions at about 531.7 eV, mainly due to the rise of the plasma induced Zn–O–H and Zn–O^−^ contributions [18]. Pristine nanocolumns are characterized by a ratio between the non-lattice oxygen (Zn–O–H, Zn–O^−^) (excluding the O=C contaminants contributions) and the lattice Zn–O–Zn structures of 0.29. Upon the H- and O-plasma treatment this ratio rises to 0.43 and 0.60, respectively. The concomitant observations in the high-resolution Zn 2p and O 1s spectra were independent from the duration of the plasma treatment. The XPS data show an increase of defect density upon the plasma treatments in the surface area of the ZnO NCs, i.e., an increased presence of Zn–O–H and Zn–O^−^ groups/species as a result of plasma seizure of the lattice Zn–O–Zn structures. The higher concentration of non-lattice oxygen (Zn–O–H and Zn–O^−^) species in the case of O-plasma treatment evidences the more effective surface activation when this type of plasma is being used.

**Figure 3:**
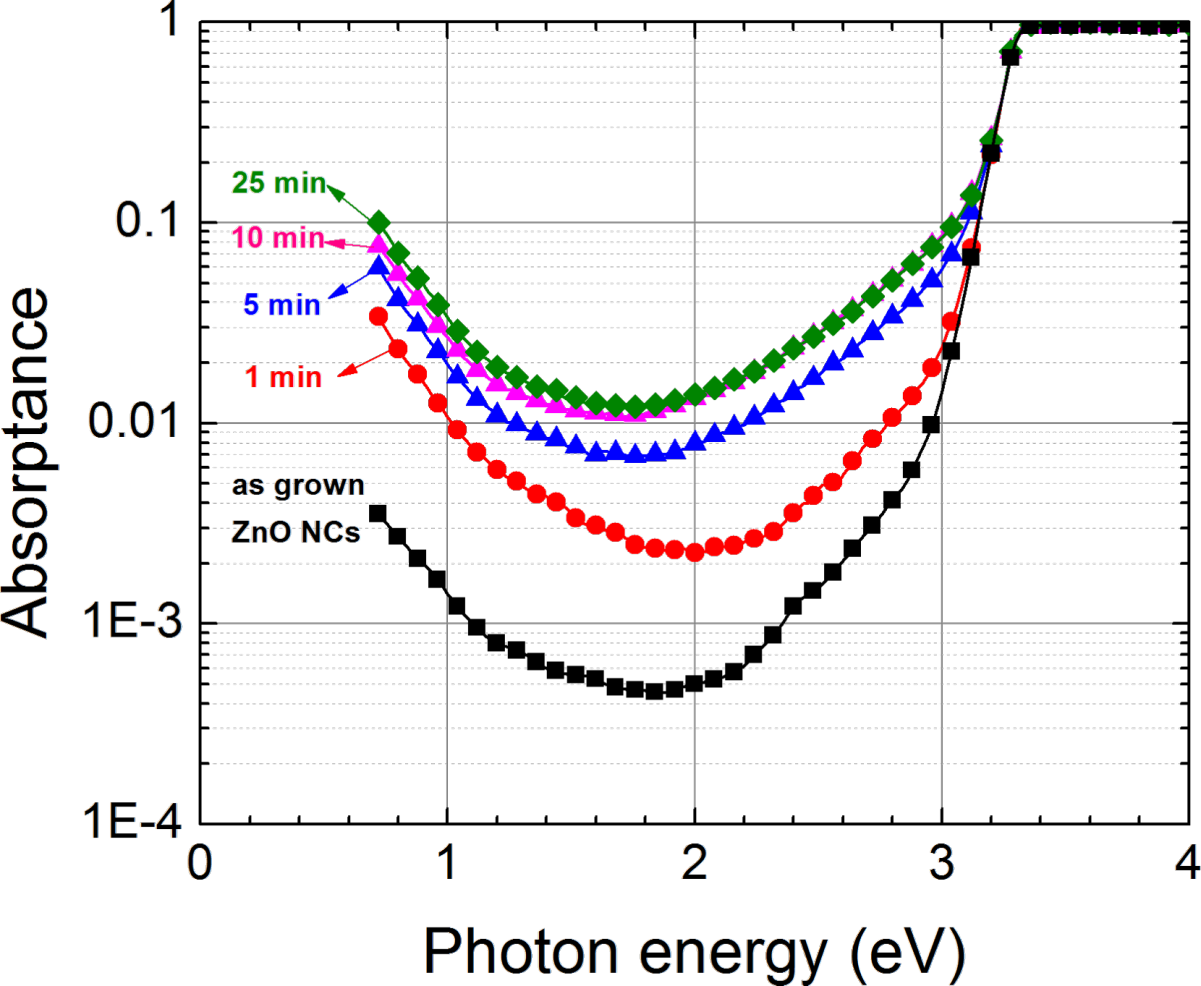
The optical absorptance spectra of as-grown ZnO nanocolumns and NCs treated in H-plasma for 1, 5, 10 and 25 min.

The changes induced to the synthesized ZnO NCs by the exposure to different plasma treatments was further probed by PDS. Figure 3 shows the optical absorptance spectra of as-grown ZnO nanocolumns and NCs treated in H-plasma for 1, 5, 10 and 25 min. The measured PDS absorptance spectra reflect the absorption edge, Urbach tail, absorption on defects and free-carrier absorption (proportional to the concentration of free carriers).

All optical absorptance spectra show the optical absorption edge at a photon energy of 3.3 eV and the free-carrier absorption in the red and the infrared part of the spectrum below photon energies of 2 eV. The infrared optical absorption increases with hydrogen plasma treatment indicating the increase of the free-carrier concentration as described in the Drude model. The increase of the free-carrier concentration is reasonably expected to increase the electrical conductivity of the ZnO NCs. Nevertheless, precise measurement of electrical conductivity is a difficult task and we plan to approach it by direct measurement on individual ZnO nanocolumns. The major changes appear within several minutes of exposure to hydrogen plasma and the effect saturates after about 10 min. We suppose that hydrogen diffuses into ZnO creating shallow donors [19–20]. We note that the hydrogen doping does not shift the optical absorption edge [21], which means that the lattice as well as the occupancy of valence and conductive states does not change significantly (up to the degenerate conduction band) [22].

**Figure 4:**
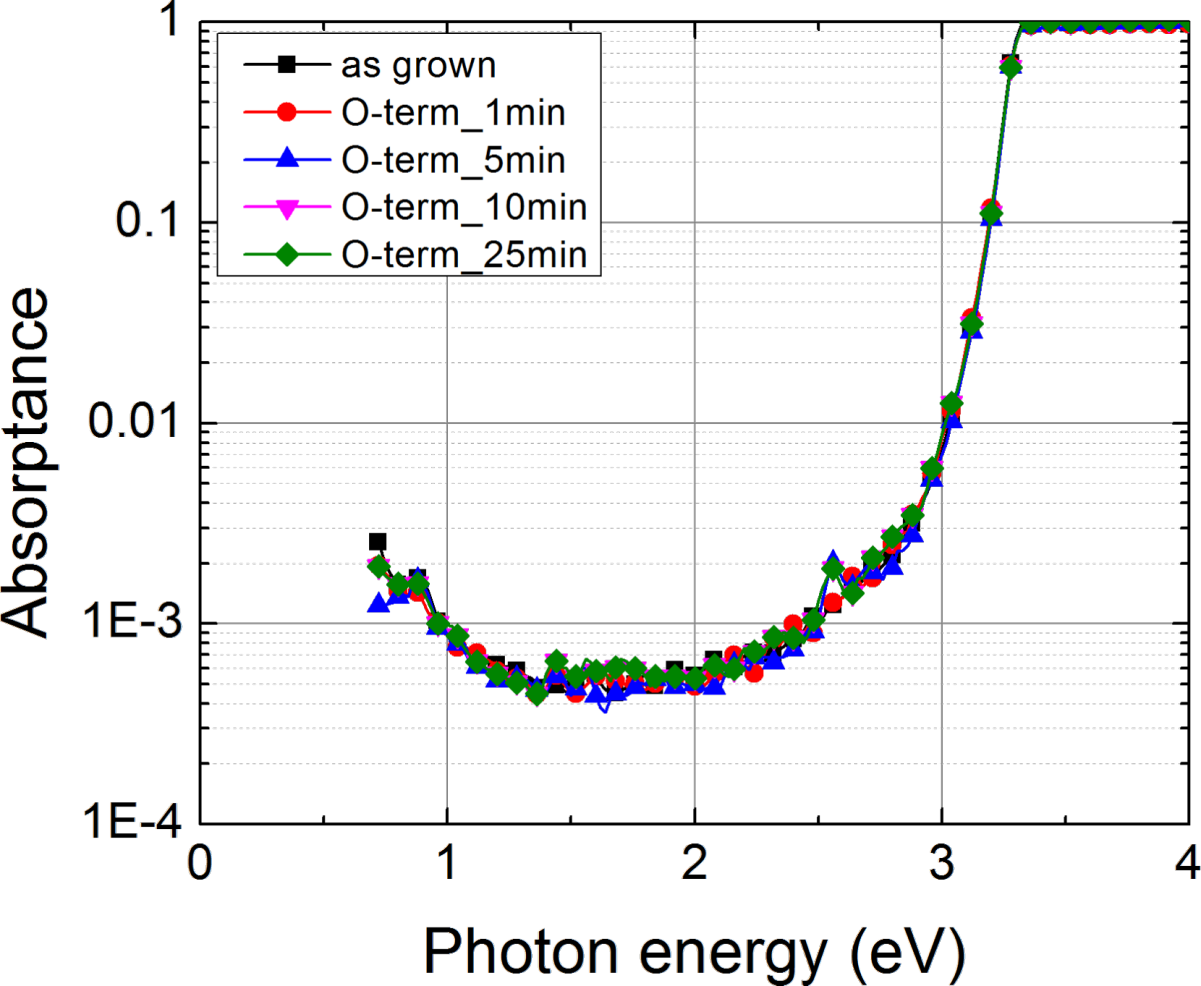
The optical absorptance spectra of as-grown ZnO nanocolumns and NCs treated in O-plasma for 1, 5, 10 and 25 min.

While the hydrogen plasma treatment induced significant changes in the optical absorptance spectra, notably, the PDS spectra show that there is no detectable change of the infrared optical absorptance (Figure 4). This observation strongly suggests that the O-plasma treatment does not have any detrimental effects on the free-carrier concentration in the ZnO NCs.

## Conclusion

In this work, randomly arranged densely packed and preferentially perpendicularly oriented ZnO nanocolumn arrays were grown from seed layers on fused silica substrates. The surface composition of the ZnO NCs drastically changes upon the exposure to H- and O-plasma treatments. The plasma treatments increase the presence of non-lattice oxygen in the form of Zn–O–H and Zn–O^−^ contributions regardless of exposure time. The O-plasma treatment led to a more effective surface activation of the ZnO NCs as evidenced by the XPS analysis. The employed hydrogen-plasma treatment led to an increase of the free-carrier adsorption of up to two orders of magnitude, thus seriously affecting the optical and photoelectrical characteristics of the ZnO NCs. The oxygen-plasma treatment led to negligible changes in the PDS absorptance spectra. The concomitant increase in the presence of active surface species and only minor influence on the optical and photoelectrical features of the ZnO NCs absorptance spectra, make the O-plasma a preferred treatment for the preparation of thin-film solar cells based on ZnO NCs. The reported ZnO nanocolumns layer with a proper spacing could be used as a 3-D scaffold not only for amorphous silicon solar cells, but also for other absorbers with a short lifetime such as CuO, CuO_2_, FeS_2_, quantum dots or nanocrystalline Si.

## Experimental

The growth of densely packed ZnO nanocolumns was performed on fused silica (Suprasil*^®^*) substrates that were covered with an undoped thin seed layer of ZnO by DC reactive magnetron sputtering. The parameters of magnetron sputtering were as follows: processing temperature of 400 °C at a target voltage of 400 V, ratio between gas species of Ar/O = 2/0.5 for 10 min.

The dimensions of all substrates were 10 × 10 mm^2^. Before seed layer deposition, the substrates were cleaned in an ultrasonic bath with acetone for 10 min, then rinsed with deionized water and dried under nitrogen flow.

The hydrothermal growth of ZnO nanocolumns was performed from an equimolar aqueous solution of 25 mmol zinc nitrate hexahydrate (Zn(NO_3_)_2_·6H_2_O) and hexamethylenetetramine ((CH_2_)_6_N_4_) in an aqueous bath at 90 °C for 3 h [15,23]. During the nanocolumns growth, the substrate was mounted upside-down on a sample holder. After termination the sample was thoroughly washed in deionized water and dried in nitrogen flow. The surface morphology of the samples was characterized by SEM (MAIA3, TESCAN). The electron energies were 5 keV or 10 keV. An in-lens detector was used to image the investigated surfaces.

### Plasma treatment

Half of the as-prepared substrates with densely packed ZnO NCs were treated in oxygen plasma at 100 W, 0.5 mbar for 1, 5, 10 and 25 min at room temperature (Femto, Diener electronic GmbH).

The other half of the substrates underwent hydrogen plasma treatment, it was done in a capacitively coupled radio frequency plasma reactor KIII [24] at 20 W, 1 mbar for 1, 5, 10 and 25 min at room temperature [25–27].

### X-ray photoelectron spectroscopy (XPS)

XPS measurements were performed using a K-Alpha^+^ XPS spectrometer (ThermoFisher Scientific, UK) operating at a base pressure of 1.0 × 10^−7^ Pa. The data acquisition and processing were performed using the Thermo Avantage software. All samples were analyzed using a microfocused, monochromated Al Kα X-ray radiation (400 µm spot size) with a pass energy of 150 eV for survey and 50 eV for high-resolution core level spectra. The X-ray angle of incidence was 30° and the emission angle was along the surface normal. The K-Alpha charge dual compensation system was employed during analysis, using electrons and low-energy argon ions to prevent any localized build-up of charge. The measured high-resolution spectra were fitted with Voigt profiles. The analyzer transmission function, Scofield sensitivity factors, and effective attenuation length for photoelectrons were applied for quantification. All spectra were referenced to the adventitious C 1s peak at a binding energy (BE) of 285.0 eV. The BE scale was controlled on standards of poly(ethylene terephthalate) and metallic Cu, Ag, and Au.

### Photothermal deflection spectroscopy (PDS)

Measurements of optical absorption on bare sputtered ZnO substrates (not shown here) as well as on as-grown densely packed ZnO NCs and further plasma-treated nanocolumns were carried out through photothermal deflection spectroscopy (PDS), a technique for measuring weak optical absorption in thin films [14,28]. Unlike optical transmittance spectroscopy, PDS detects directly the amount of the absorbed light by measuring the heat generated by the optical absorption with the optical absorptance sensitivity down to 0.01%. PDS is based on measuring the deflection of the laser beam in the vicinity of the thin film immersed in a transparent liquid due to thermal changes of medium induced by heating of thin film by the absorption of the incident monochromatic light. A significant advantage of the PDS is the weak influence of the optical scattering, which is important in the case of ZnO nanocolumns for which the transmittance spectra are significantly deteriorated by optical scattering.

## Supporting Information

File 1 Additional SEM pictures.

## References

[1] Krishnakumar V, Han J, Klein A, Jaegermann W (2011). Thin Solid Films.

[2] Bosio A, Menossi D, Mazzamuto S, Romeo N (2011). Thin Solid Films.

[3] Shah A, Moulin E, Ballif C (2013). Sol Energy Mater Sol Cells.

[R4] 4Lin, J.; Cashmore, J.; Iwahashi, T.; Sakurai, J.; Losio, P.; Orhan, J. B.; Hoetzel, J.; Ristau, S.; Saito, Y.; Tsuji, A.; Roschek, T.; Meier, J.; Sinicco, I.; Zindel, A.; Hayashi, H.; Matsunaga, D. *Blazing New Paths to High Efficiency Amorphous Silicon Based Solar Modules.* Oral presentation at 40th IEEE Photovoltaics Specialist Conference, Denver, CO, USA, June 8–13, 2014. http://www.flip-programs.com/PVSC/PVSC-40-YB/

[5] Vanecek M, Babchenko O, Purkrt A, Holovsky J, Neykova N, Poruba A, Remes Z, Meier J, Kroll U (2011). Appl Phys Lett.

[6] Neykova N, Moulin E, Campa A, Hruska K, Poruba A, Stuckelberger M, Haug F-J, Topic M, Ballif C, Vanecek M (2015). Phys Status Solidi A.

[7] Vanecek M, Neykova N, Hruska K, Poruba A, Remes Z, Holovsky J, Purkrt A, Babchenko O, Meier J, Kroll U (2014). Photovoltaic cell and methods for producing a photovoltaic cell. U.S. Patent.

[8] Kim J, Battaglia C, Charrière M, Hong A, Jung W, Park H, Ballif C, Sadana D (2014). Adv Mater.

[9] Kuang Y, van der Werf K H M, Houweling Z S, Schropp R E I (2011). Appl Phys Lett.

[10] Ho C-I, Liang W-C, Yeh D-J, Su V-C, Yang P-C, Chen S-Y, Yang T-T, Lee J-H, Kuan C-H, Cheng I-C (2013). J Appl Phys.

[11] Liu X-Y, Shan C-X, Wang S-P, Zhao H-F, Shen D-Z (2013). Nanoscale.

[12] Elias J, Tena-Zaera R, Lévy-Clément C (2008). J Electroanal Chem.

[13] Mamat M H, Malek M F, Hafizah N N, Asiah M N, Suriani A B, Mohamed A, Nafarizal N, Ahmad M K, Rusop M (2016). Ceram Int.

[14] Neykova N, Brož A, Remeš Z, Hruška K, Kalbáčová M, Kromka A, Vaněček M (2012). Appl Surf Sci.

[15] Neykova N, Hruska K, Holovsky J, Remes Z, Vanecek M (2013). Thin Solid Films.

[16] Jackson W B, Amer N M, Boccara A C, Fournier D (1981). Appl Opt.

[17] Granqvist C G, Hultåker A (2002). Thin Solid Films.

[18] Chen C, Lu Y, He H, Xiao M, Wang Z, Chen L, Ye Z (2013). ACS Appl Mater Interfaces.

[19] Cox S F J, Davis E A, Cottrell S P, King P J C, Lord J S, Gil J M, Alberto H V, Vilão R C, Piroto Duarte J, Ayres de Campos N (2001). Phys Rev Lett.

[20] Hofmann D M, Hofstaetter A, Leiter F, Zhou H, Henecker F, Meyer B K, Orlinskii S B, Schmidt J, Baranov P G (2002). Phys Rev Lett.

[21] Miloslavskii V K, Pogrebniak P S (1972). Phys Status Solidi B.

[22] Kim Y H, Karazhanov S Zh, Kim W M (2011). Phys Status Solidi B.

[23] Greene L E, Law M, Goldberger J, Kim F, Johnson J C, Zhang Y, Saykally R J, Yang P (2003). Angew Chem, Int Ed.

[24] Stuchlik J, Stuchlikova T H, Artemenko A, Remes Z (2015). JJAP Conf Proc.

[25] Neykova N, Kozak H, Ledinsky M, Kromka A (2012). Vacuum.

[26] Pop-Georgievski O, Neykova N, Proks V, Houdkova J, Ukraintsev E, Zemek J, Kromka A, Rypaček F (2013). Thin Solid Films.

[27] Krátká M, Neykova N, Ukraintsev E, Kromka A, Rezek B (2013). Int J Electrochem Sci.

[28] Remes Z, Babchenko O, Varga M, Stuchlik J, Jirasek V, Prajzler V, Nekvindova P, Kromka A (2016). Thin Solid Films.

