# Supplementary material for: Study of the surface properties of ZnO nanocolumns used for thin-film solar cells
Source: Beilstein J Nanotechnol. 2017 Feb 16;8:446–51. doi: 10.3762/bjnano.8.48 (PMC5331301; doi:10.3762/bjnano.8.48)
Supplement: File 1 — Additional SEM pictures. [file Beilstein_J_Nanotechnol-08-446-s001.pdf]

Supporting Information

for

Study of the surface properties of ZnO nanocolumns used for thin-film solar cells

Neda Neykova*[1], Jiri Stuchlik[1], Karel Hruska[1], Ales Poruba[1], Zdenek Remes[1] and Ognen Pop-Georgievski[2]

Address: [1]Institute of Physics AS CR v.v.i., Cukrovarnicka 10, 162 53 Prague, Czech Republic and [2]Institute of Macromolecular Chemistry AS CR v.v.i., Heyrovsky sq. 2, 162 06 Prague, Czech Republic

Email: Neda Neykova* - neykova@fzu.cz

* Corresponding author

Additional SEM pictures

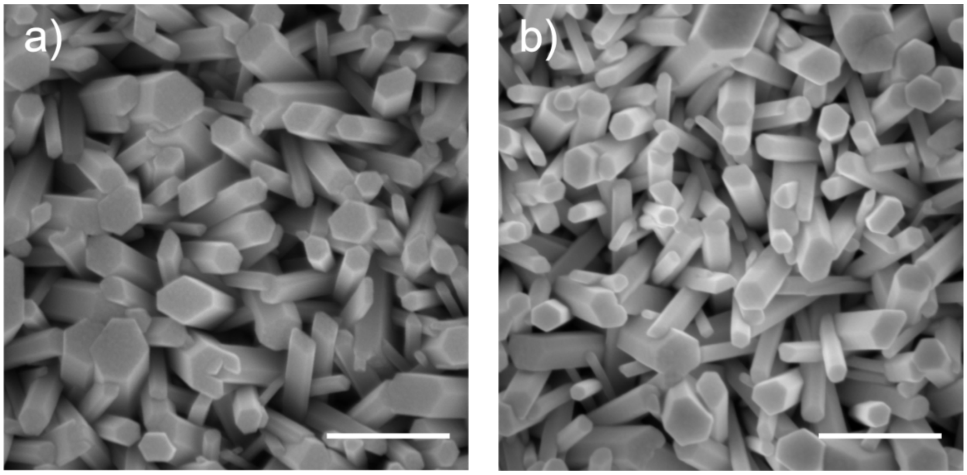

Figure S1: SEM micrographs of ZnO nanocolumns before (a) and after treatment in hydrogen plasma for 25 min (scale bar of 500 nm).

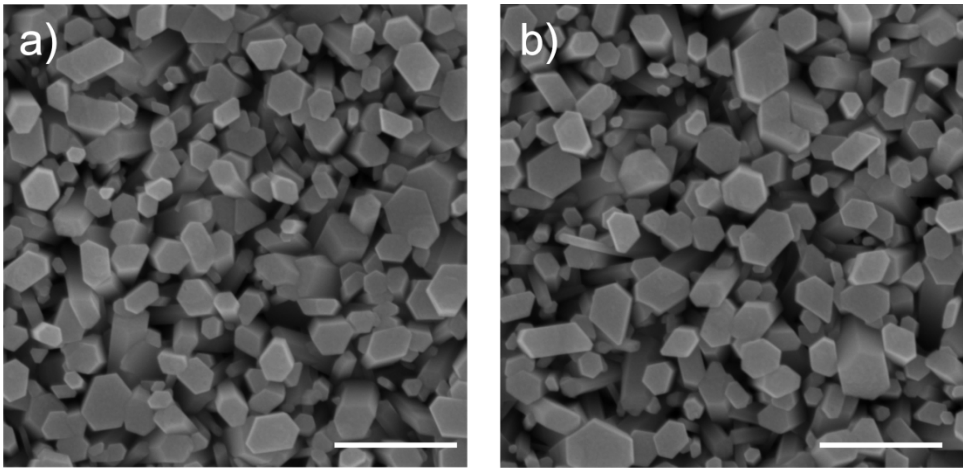

Figure S2: SEM micrographs of ZnO nanocolumns before (a) and after treatment in oxygen plasma for 25 min (scale bar of 500 nm).
